# Changing trends in cancer incidence of upper aerodigestive tract and stomach in Japanese alcohol‐dependent men (1993‐2018)

**DOI:** 10.1002/cam4.2737

**Published:** 2019-12-03

**Authors:** Akira Yokoyama, Tai Omori, Tetsuji Yokoyama

**Affiliations:** ^1^ National Hospital Organization Kurihama Medical and Addiction Center Kanagawa Japan; ^2^ Endoscopy Center Kawasaki Municipal Ida Hospital Kanagawa Japan; ^3^ Department of Health Promotion National Institute of Public Health Saitama Japan

**Keywords:** alcohol dependence, aldehyde dehydrogenase‐2, chronic atrophic gastritis, esophageal cancer, gastric cancer, head and neck cancer

## Abstract

**Background:**

Esophageal squamous cell carcinoma (ESCC), head and neck SCC (HNSCC), and gastric adenocarcinoma (GA) are frequently detected at an early stage using endoscopic screening in Japanese alcohol‐dependent men.

**Methods:**

We performed endoscopic screening with esophageal iodine staining and oropharyngolaryngeal inspection in 7582 Japanese alcohol‐dependent men (40‐79 years) during 1993‐2018, and retrospectively investigated their initial screening results.

**Results:**

The 2008‐2018 screening showed lower detection rates for ESCC (2.6% vs 4.0%, *P* = .0009) and GA (0.5% vs 1.4%, *P* < .0001) for all age brackets, compared with the 1993‐2007 screening. The HNSCC detection rate did not change (1.0% vs 1.1%). Multiple logistic regression analyses showed that the 2008‐2018 screening had a reduced OR (95% CI) for ESCC (0.34 [0.25‐0.47]) and GA (0.19 [0.10‐0.35]), compared with the 1993–2007 screening. The reduction in *H pylori* infection is probably the main reason for the decrease in GA detection over time. Declining trends in pack‐years and gastric atrophy and increasing trends in age and body mass index (BMI) were found over time. The presence of advanced gastric atrophy increased the risk for ESCC as well as GA. The inactive heterozygous aldehyde dehydrogenase‐2*1/*2 genotype was a strong risk factor for ESCC, HNSCC, and GA. Fewer pack‐years and a larger BMI decreased the ESCC risk. However, these confounders cannot fully explain why the incidence of ESCC has decreased markedly over the recent decade.

**Conclusions:**

The detection rates of ESCC and GA have markedly decreased during the past decade in the alcohol‐dependent population. The enigmatic declining trend of ESCC warrants research on this topic.

## Graphical abstract

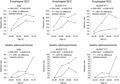

## 1 INTRODUCTION

Esophageal squamous cell carcinoma (ESCC), head and neck squamous cell carcinoma (HNSCC) and gastric adenocarcinoma (GA) are frequently detected at an early stage using endoscopic screening in Japanese alcohol‐dependent men.^1^, ^2^ Several risk factors for these cancers have been identified in this population including age, drinking and smoking habits,^3^ body mass index (BMI),^3^ genetic polymorphisms of *aldehyde dehydrogenase‐2* (*ALDH2*; rs671) and *alcohol dehydrogenase‐1B* (*ADH1B*; rs1229984),^1^, ^2^, ^3^, ^4^ and *Helicobacter pylori* (*H pylori*)‐associated gastric atrophy.^5^, ^6^ Aging, changes in popular alcohol‐beverage types,^7^ less smoking,^8^ an increase in BMI,^9^ and a declining trend in the *H pylori* infection rate^10^ have occurred in Japan during the recent decades. This study evaluated the changes in the risk factors and detection rates of ESCC, HNSCC, and GA among Japanese alcohol‐dependent men who underwent endoscopic screening during 1993‐2018.

## 2 MATERIALS AND METHODS

### 2.1 Subjects

The reference population included 8677 Japanese alcohol‐dependent men aged 40‐79 years who visited the Kurihama Medical and Addiction Center for treatment of alcohol dependence and who underwent routine upper gastrointestinal endoscopic screening with esophageal iodine staining and oropharyngolaryngeal inspection between 1993 and 2018. Some patients developed cancer during follow‐up screening, but we used the results of their initial screening in this study and there was no overlap among the patients.

A history of esophageal cancer treatment was found in 94 patients (1.1%; treated with surgery in 65; chemoradiation in 11; and endoscopic mucosectomy in 18). A history of head and neck cancer treatment was found in 51 patients (0.6%; treated with surgery in 29; chemoradiation in 16; endoscopic mucosectomy in 3; and unknown in 3). A history of gastric cancer treatment was found in 376 patients (4.3%; treated with surgery in 357 and endoscopic mucosectomy in 19). A history of gastrectomy was found in 963 patients (11.1%; treated for peptic ulcer in 592, gastric cancer in 357, and other causes in 14). After excluding 1095 patients with any history of the cancer treatment or a gastrectomy, 7582 patients were included in this study.

All the subjects met the DSM‐IIIR, DSM‐IV, or ICD‐10 criteria for alcohol dependence.^11^, ^12^, ^13^ Each subject was asked about his drinking and smoking habits using a structured questionnaire, as previously reported.^1^, ^2^ The proposal for this study was approved by the ethics committee of the Kurihama Medical and Addiction Center. All records were obtained as anonymized data. The ethics committee determined that the requirement for additional informed consent to participate in this study was waived due to its retrospective design, and patients could exclude themselves by using the opt‐out method on the Center's website.

### 2.2 Endoscopic screening

Examinations were performed using Olympus endoscopes (models Q10, P20, XQ200, XQ230, Q240, Q240Z, Q260, and Q260Z in chronological order of use; Olympus Optical Co. Ltd.). Almost all the screening was performed by a single endoscopist (A. Yokoyama) or was performed under his supervision. The screening program and diagnostic procedure have been described in previous reports.^1^, ^2^ The routine application of narrow band imaging (NBI) for inspection of the upper aerodigestive tract was begun in 2009.

### 2.3 Chronic atrophic gastritis (CAG)

As reported in our earlier paper,^6^ the serum pepsinogen (PG) levels were measured in 90 ESCC patients diagnosed between 1993 and 2002 and 180 age‐matched control patients between 2000 and 2002. Serological CAG was diagnosed based on the criteria for a positive PG test.^6^, ^14^, ^15^, ^16^

Using digitalized gastric images stored within a medical imaging communication system since 2003, a single endoscopist (A. Yokoyama) reviewed the endoscopic findings for CAG according to the Kimura‐Takemoto classification system.^17^ The patients were classified into three categories (C0 to C2, C3 to O1, and O2‐O3) because the gastric cancer detection rate reportedly increases in a stepwise manner according to these categories.^18^

### 2.4 *ALDH2* and *ADH1B* genotyping

We previously determined the *ALDH2* and *ADH1B* genotypes of 5630 subjects from whom written informed consent had been obtained for the study of *ADH1B* and *ALDH2* genotype‐associated phenotypes and comorbidities which had been approved by the ethics committee of the Center. PCR‐restriction fragment length polymorphism methods were used to genotype *ALDH2* and *ADH1B* in DNA obtained from blood samples.^1^

### 2.5 Statistical analysis

Values were expressed as mean and standard deviation (SD) or in percentage. *P* values for categorical data were calculated using the Chi‐square test for homogeneity or the Cochran‐Mantel‐Haenszel test for trend, where appropriate. The general linear model was used to test for trends of mean values across groups. Detection rates of cancer were compared between study periods adjusted for age using the Cochran‐Mantel‐Haenszel test. The multivariate odds ratios (ORs) and the 95% confidence intervals (CIs) were calculated using multiple logistic models. We combined the ADH1B*1/*2 genotype carriers and the ADH1B*2/*2 genotype carriers into a group because of the semidominant nature of the ADH1B*2 allele.^19^, ^20^ Statistical significance was defined as a *P* value <.05. All the statistical analyses were performed using the SAS software program (version 9.4; SAS Institute).

## 3 RESULTS

The initial screening of the 7582 patients indicated ESCC in 262 (3.5%) patients, HNSCC in 82 (1.1%; 18 in the oral cavity, 21 in the oropharynx, 47 in the hypopharynx, and nine in the larynx), and gastric adenocarcinoma (GA) in 80 (1.1%; five in the gastric cardia alone, 72 in the gastric noncardia alone, and three in both sites). Concurrent ESCC and HNSCC were found in 39 patients, concurrent ESCC and GA were found in 11, and concurrent HNSCC and GA were found in six. Three patients had concurrent ESCC, HNSCC, and GA. One case each of Barrett's adenocarcinoma, esophageal melanoma, gastric lymphoma, and gastric carcinoid, two cases of duodenal adenocarcinoma, and 376 (5.0%) cases of any type of cancer were found.

Table 1 shows the background factors. The age distributions shifted toward higher age ranges over time, and the mean age increased by 2‐3 years. Although the increase in usual alcohol consumption during the preceding year reached a significant level, the ranges were between 111 and 120 g ethanol/day. The most frequently consumed alcoholic beverages during the preceding year changed over time, with sake and whiskey decreasing and shochu, wine, and canned chuhai increasing. The numbers of never‐ and ex‐smokers increased. Pack‐years progressively decreased by 7.5 pack‐years. BMI progressively increased by about 1 kg/m^2^. A decreasing trend of severe CAG was found. No significant changes in the *ALDH2* and *ADH1B* genotypes were observed.

**Table 1:** Changes in background factors between 1993 and 2018

	Year of endoscopic screening					*P*
	1993‐1997	1998‐2002	2003‐2007	2008‐2012	2013‐2018	
Age (y)	N = 1631	1544	1597	1539	1271	
40‐49	34.8%	32.6%	29.5%	31.3%	31.7%	<.0001
50‐59	42.0%	39.4%	37.6%	31.1%	36.8%	
60‐69	20.0%	22.2%	25.4%	27.0%	21.2%	
70‐79	3.2%	5.8%	7.5%	10.7%	10.3%	
Mean ± SD	53.5 ± 8.0	54.4 ± 8.6	55.3 ± 9.1	56.1 ± 9.9	55.4 ± 9.7	<.0001
Usual alcohol intake (g ethanol/day)	N = 1631	1544	1597	1539	1271	
Mean ± SD	115.3 ± 63.0	110.9 ± 63.0	116.4 ± 71.3	119.8 ± 75.5	117.9 ± 74.7	.014
Alcoholic beverage most frequently consumed	N = 1599	1538	1595	1538	1271	
Beer/low‐malt beer (4%‐5% ethanol v/v)	9.9%	12.9%	11.7%	14.2%	12.4%	<.0001
Canned chuhai (4%‐9%)	1.8%	4.7%	7.0%	7.7%	16.8%	
Wine (12%)	0.3%	1.2%	1.1%	1.1%	1.7%	
Sake (15%‐16%)	39.9%	28.4%	23.4%	18.1%	13.5%	
Shochu (20%‐25%)	30.6%	37.1%	43.5%	50.6%	44.8%	
Whiskey/other spirits (40%)	17.5%	15.6%	13.2%	8.2%	10.8%	
Cigarette smoking	N = 1602	1536	1597	1539	1271	
Never	6.9%	8.3%	10.6%	8.7%	11.6%	<.0001
1‐19 cigs/day	23.2%	20.8%	24.6%	24.4%	27.5%	
20 + cigs/day	63.5%	61.4%	55.3%	50.9%	40.0%	
Ex‐smoker	6.4%	9.5%	9.5%	15.9%	21.0%	
Pack‐years (mean ± SD)	35.9 ± 23.8	35.9 ± 24.0	33.4 ± 23.8	32.3 ± 22.9	28.4 ± 23.6	<.0001
Body mass index (kg/m^2^)	N = 1547	1501	1565	1486	1248	
<18.5	20.3%	17.1%	15.0%	15.9%	14.5%	<.0001
18.5‐24.9	69.5%	69.0%	68.4%	66.6%	66.3%	
≧25	10.2%	14.0%	16.6%	17.4%	19.2%	
Mean ± SD	21.1 ± 3.0	21.5 ± 3.3	21.8 ± 3.4	21.9 ± 3.6	22.1 ± 3.5	<.0001
Chronic atrophic gastritis†	—	—	N = 1319	1533	1264	<.0001
C0 to C2	—	—	59.9%	64.1%	75.6%	
C3 to O1	—	—	27.6%	25.4%	17.9%	
O2 to O3	—	—	12.5%	10.5%	6.5%	
*ALDH2* genotype	N = 751	1128	1128	1426	1197	
*1/*1 (active)	85.0%	85.4%	85.6%	85.1%	82.9%	.17
*1/*2 (inactive)	15.0%	14.6%	14.4%	14.9%	17.1%	
*ADH1B* genotype	N = 751	1128	1128	1426	1197	
*1/*1 (slow metabolizing)	31.3%	30.5%	27.8%	28.6%	28.0%	.074
*1/*2 (fast metabolizing)	30.4%	35.8%	35.0%	33.5%	33.6%	
*2/*2 (fast metabolizing)	38.3%	33.7%	37.1%	37.9%	38.4%	

Note

*P* values are for the Chi‐square test for cigarette smoking, the Cochran‐Mantel‐Haenszel test for other categorical data, and regression analyses for the mean values.

Abbreviations: ADH1B, alcohol dehydrogenase‐1B gene; ALDH2, aldehyde dehydrogenase‐2 gene.

† Kimura‐Takemoto classification for endoscopic gastric atrophy.

The ESCC detection rate increased from the 1993‐1997 period (3.7%) to the 2003‐2007 period (4.3%) and decreased during the 2008‐2012 period (3.0%) and the 2013‐2018 period (2.1%) (*P* for trend = .013; Table 2). When the ESCC detection rate during 2003‐2007 was used as the reference, the age‐adjusted OR for ESCC detection decreased (*P* for trend = .001) during the 2008‐2012 screening (OR [95% CI] = 0.65 [0.44‐0.95]) and the 2013‐2018 screening (0.47 [0.30‐0.74]). The detection rate for GA gradually decreased over time, but the HNSCC detection rate did not change significantly.

**Table 2:** Changes in cancer detection rates between 1993 and 2018

	Year of endoscopic screening					
	1993‐1997	1998‐2002	2003‐2007	2008‐2012	2013‐2018	*P*
	(N = 1631)	(N = 1544)	(N = 1597)	(N = 1539)	(N = 1271)	
ESCC						
Detection rate	3.7%	3.9%	4.3%	3.0%	2.1%	.013
Age‐adjusted OR (95% CI)	0.93 (0.65‐1.33)	0.94 (0.66‐1.34)	1 (referent)	0.65 (0.44‐0.95)	0.47 (0.30‐0.74)	.001
HNSCC						
Detection rate	0.8%	1.2%	1.4%	1.2%	0.9%	.76
Age‐adjusted OR (95% CI)	0.60 (0.30‐1.20)	0.86 (0.46‐1.62)	1 (referent)	0.83 (0.44‐1.56)	0.62 (0.30‐1.29)	.90
GA						
Detection rate	1.5%	1.4%	1.3%	0.3%	0.7%	.0006
Age‐adjusted OR (95% CI)	1.49 (0.82‐2.70)	1.25 (0.68‐2.31)	1 (referent)	0.18 (0.06‐0.54)	0.53 (0.24‐1.17)	<.0001

Note

*P* values were calculated using the Cochran‐Mantel‐Haenszel test for trends in percentage data and logistic regression analyses for odds ratios.

Abbreviations: CI, confidence interval; ESCC, esophageal squamous cell carcinoma; GA, gastric adenocarcinoma; HNSCC, head and neck squamous cell carcinoma; OR, odds ratio.

The cancer invasion depth for ESCC and GA did not change during the study period, but a decreasing trend for the invasion of SCC to the proper muscle layer or beyond and an increasing trend toward the confinement of SCC to the epithelium were observed for HNSCC (*P* = .079; Table 3).

**Table 3:** Trends in cancer invasion depth between 1993　and 2018

Cancer Invasion Depth	Year of endoscopic screening					*P*
	1993‐1997	1998‐2002	2003‐2007	2008‐2012	2013‐2018	
ESCC						
Intraepithelium	23 (38.3%)	17 (28.3%)	35 (50.7%)	18 (39.1%)	13 (48.1%)	.39
Proper mucosal layer	15 (25.0%)	16 (26.7%)	13 (18.8%)	9 (19.6%)	6 (22.2%)	
Muscularis mucosa	7 (11.7%)	10 (16.7%)	7 (10.1%)	8 (17.4%)	3 (11.1%)	
Submucosa	9 (15.0%)	13 (21.7%)	9 (13.0%)	4 (8.7%)	3 (11.1%)	
Beyond proper muscle layer	6 (10.0%)	4 (6.7%)	5 (7.2%)	7 (15.2%)	2 (7.4%)	
HNSCC						
Intraepithelium	2 (15.4%)	5 (27.8%)	6 (27.3%)	6 (33.3%)	4 (36.4%)	.079
Subepithelium	7 (53.8%)	9 (50.0%)	13 (59.1%)	10 (55.6%)	6 (54.5%)	
Beyond proper muscle layer	4 (30.8%)	4 (22.2%)	3 (13.6%)	2 (11.1%)	1 (9.1%)	
GA						
Mucosal layer	8 (32.0%)	10 (45.5%)	10 (50.0%)	2 (50.0%)	4 (44.4%)	.63
Submucosal layer	12 (48.0%)	7 (31.8%)	8 (40.0%)	0 (0.0%)	3 (33.3%)	
Beyond proper muscle layer	5 (20.0%)	5 (22.7%)	2 (10.0%)	2 (50.0%)	2 (22.2%)	

Note

*P* values were calculated using the Cochran‐Mantel‐Haenszel test for trends.

Abbreviations: ESCC, esophageal squamous cell carcinoma; GA, gastric adenocarcinoma; HNSCC, head and neck squamous cell carcinoma.

**Figure 1:**
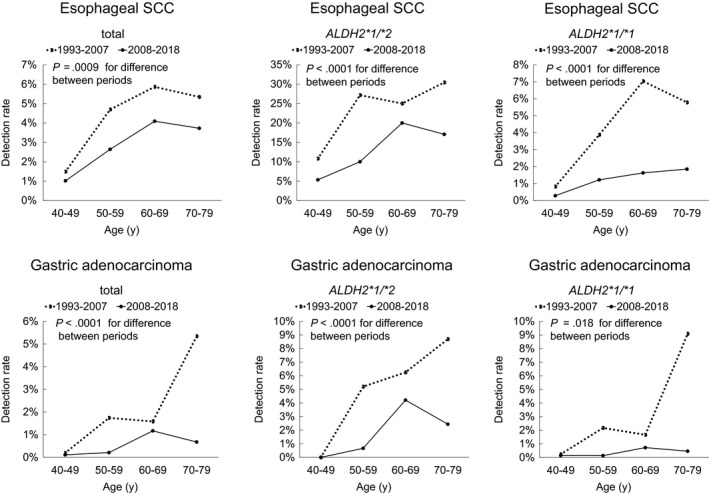
Comparison of detection rates of esophageal squamous cell carcinoma (ESCC) and gastric adenocarcinoma (GA) according to age groups and *ALDH2* genotype between the 1993‐2007 screening period and the 2008‐2018 screening period in Japanese alcohol‐dependent men. The detection rates for ESCC and GA were consistently lower during the 2008‐2018 study period than during the 1993‐2007 study period regardless of age groups and *ALDH2* genotype

The cancer detection rates for the 1993‐2007 screening and 2008‐2018 screening were 4.0% and 2.6%, respectively, for ESCC, 1.1% and 1.0%, respectively, for HNSCC, and 1.4% and 0.5%, respectively, for GA. The rates of using esophageal iodine staining were 93.3% and 92.3%, respectively. Figure 1 shows a comparison of the detection rates of ESCC and GA between the 1993‐2007 screening and the 2008‐2018 screening according to age groups and *ALDH2* genotype. The detection rates for ESCC and GA were consistently lower during the 2008‐2018 period than during the 1993‐2007 period regardless of age groups and *ALDH2* genotype.

Multiple logistic regression analyses showed that an older age increased the ORs for ESCC, HNSCC, and GA (Table 4). The ORs (95% CI) for ESCC decreased for the 2008‐2018 period, compared with the 1993‐2007 period (0.34 [0.25‐0.47]) and per 1 kg/m^2^ increment of BMI (0.93 [0.89‐0.97]) and increased per 10 pack‐years increment (1.06 [1.01‐1.12]), for the ALDH2*1/*2 genotype (8.49 [6.47‐11.13]), and for the ADH1B*1/*1 genotype (2.42 [1.83‐3.20]). The ORs for HNSCC increased for the ALDH2*1/*2 genotype (6.82 [4.29‐10.86]) and for the ADH1B*1/*1 genotype (2.84 [1.77‐4.55]). The ORs for GA decreased for the 2008‐2018 period, compared with the 1993‐2007 period (0.19 [0.10‐0.35]) and increased for the ALDH2*1/*2 genotype (2.59 [1.56‐4.30]). The type of alcoholic beverages most frequently consumed did not affect the ORs for ESCC, HNSCC, or GA.

**Table 4:** Multiple logistic regression analyses for risk factors of cancer in the esophagus, head and neck, and stomach (1993‐2018)

	ESCC	HNSCC	GA
	OR (95% CI)	OR (95% CI)	OR (95% CI)
Age (per + 10 y)	1.79 (1.53‐2.10)	1.33 (1.01‐1.75)	2.39 (1.82‐3.14)
Usual alcohol intake (per + 22 g ethanol/day)	0.97 (0.92‐1.02)	0.96 (0.87‐1.05)	1.05 (0.98‐1.12)
Alcoholic beverage most frequently consumed			
Beer/low‐malt beer/canned chuhai	1 (referent)	1 (referent)	1 (referent)
Sake/wine	1.11 (0.72‐1.71)	0.87 (0.38‐1.97)	1.47 (0.61‐3.53)
Shochu/whiskey/other spirits	1.36 (0.93‐2.00)	1.73 (0.90‐3.33)	1.88 (0.84‐4.22)
Pack‐years (per + 10 pack‐year)	1.06 (1.01‐1.12)	1.09 (0.99‐1.19)	1.06 (0.98‐1.15)
BMI (per + 1 kg/m^2^)	0.93 (0.89‐0.97)	0.94 (0.87‐1.01)	1.00 (0.93‐1.08)
Year of screening (2008‐2018 vs 1993‐2007)	0.34 (0.25‐0.47)	0.61 (0.37‐1.002)	0.19 (0.10‐0.35)
ALDH2*1/*2 carriers vs *1/*1 carriers	8.49 (6.47‐11.13)	6.82 (4.29‐10.86)	2.59 (1.56‐4.30)
ADH1B*1/*1 carriers vs *2 carriers	2.42 (1.83‐3.20)	2.84 (1.77‐4.55)	0.76 (0.43‐1.35)

Note

ORs were estimated using multiple logistic regression models with all variables entered into the model.

Abbreviations: *ADH1B*, alcohol dehydrogenase‐1B gene; *ALDH2*, aldehyde dehydrogenase‐2 gene; BMI, body mass index; CI, confidence interval; OR, odds ratio; SCC, squamous cell carcinoma.

We calculated the ORs for ESCC according to the serological CAG in the 1993‐2002 screening group and the endoscopical CAG in the 2003‐2018 screening group (Table 5). Multiple regression analyses showed increased ORs for ESCC according to the presence of severe serological CAG (5.58 [2.46‐12.66]) and severe endoscopical CAG (2.46 [1.45‐4.19]).

**Table 5:** Multiple logistic regression analyses for risk factors, including atrophic gastritis, for ESCC (1993‐2018)

		1993‐2002	2003‐2018
		OR (95% CI)	OR (95% CI)
Age (per + 10 y)		0.94 (0.56‐1.57)§	1.59 (1.26‐2.00)
Usual alcohol intake (per + 22 g ethanol/day)		1.08 (0.97‐1.21)	0.97 (0.91‐1.05)
Alcoholic beverage most frequently consumed			
Beer/low‐malt beer/canned chuhai		1 (referent)	1 (referent)
Sake/wine		1.16 (0.43‐3.13)	1.36 (0.73‐2.54)
Shochu/whiskey/other spirits		0.85 (0.34‐2.12)	1.39 (0.82‐2.35)
Pack‐years (per + 10 pack‐year)		0.99 (0.87‐1.12)	1.05 (0.97‐1.13)
BMI (per + 1 kg/m^2^)		0.82 (0.74‐0.92)	0.93 (0.87‐0.99)
Chronic atrophic gastritis			
Serum PG test†	Endoscopic atrophy‡		
Negative	C0‐C2	1 (referent)†	1 (referent)‡
Positive, nonsevere	C3‐O1	1.04 (0.46‐2.33)	1.53 (0.96‐2.42)
Positive, severe	O2‐O3	5.58 (2.46‐12.66)	2.46 (1.45‐4.19)
ALDH2*2 carriers vs *1/*1 carriers		10.24 (5.09‐20.60)	9.17 (6.24‐13.47)
ADH1B*1/*1 carriers vs *2 carriers		3.02 (1.52‐5.99)	2.94 (1.97‐4.38)

Abbreviations: *ADH1B*, alcohol dehydrogenase‐1B gene; *ALDH2*, aldehyde dehydrogenase‐2 gene; BMI, body mass index; CI, confidence interval; ESCC, esophageal squamous cell carcinoma; OR, odds ratio; PG, pepsinogen.

ORs were estimated using multiple logistic regression models with all the variables entered into the model.

^†^ The reanalysis was done using the data reported in Ref. [6].

^‡^ Kimura‐Takemoto classification for endoscopic gastric atrophy

^§^ The controls were frequency‐matched for 5‐year age groups.

Endoscopical CAG markedly increased the OR for GA (9.24 [2.53‐33.75] for C3‐O1 atrophy; 19.78 [5.25‐74.55] for O2‐O3 atrophy; Table 6). The ALDH2*1/*2 genotype increased the ORs for GA (2.52 [1.07‐5.92]) in this logistic model including CAG. CAG was not associated with the risk of HNSCC.

**Table 6:** Multiple logistic regression analyses for risk factors, including atrophic gastritis, of cancer in the head and neck and in the stomach (2003‐2018)

	HNSCC	GA
	OR (95% CI)	OR (95% CI)
Age (per + 10 y)	1.31 (0.92‐1.85)	1.38 (0.87‐2.20)
Usual alcohol intake (per + 22 g ethanol/day)	1.00 (0.90‐1.10)	1.09 (1.02‐1.17)
Alcoholic beverage most frequently consumed		
Beer/low‐malt beer/canned chuhai	1 (referent)	1 (referent)
Sake/wine	0.99 (0.32‐3.05)	6.95 (0.85‐57.09)
Shochu/whiskey/other spirits	2.06 (0.89‐4.77)	5.38 (0.70‐41.13)
Pack‐years (per + 10 pack‐year)	1.04 (0.92‐1.18)	1.05 (0.92‐1.20)
BMI (per + 1 kg/m^2^)	0.92 (0.84‐1.01)	0.92 (0.81‐1.04)
Chronic atrophic gastritis†		
C0 to C2	1 (referent)	1 (referent)
C3 to O1	1.10 (0.54‐2.24)	9.24 (2.53‐33.75)
O2 to O3	1.08 (0.42‐2.78)	19.78 (5.25‐74.55)
ALDH2*1/*2 carriers vs *1/*1 carriers	7.09 (3.95‐12.74)	2.52 (1.07‐5.92)
ADH1B*1/*1 carriers vs *2 carriers	2.70 (1.49‐4.90)	0.69 (0.23‐2.06)

Note

ORs were estimated using multiple logistic regression models with all the variables entered into the model.

Abbreviations: *ADH1B*, alcohol dehydrogenase‐1B gene; *ALDH2*, aldehyde dehydrogenase‐2 gene; BMI, body mass index; CI, confidence interval; GA, gastric adenocarcinoma; HNSCC, head and neck squamous cell carcinoma; OR, odds ratio.

^†^ Kimura‐Takemoto classification for endoscopic gastric atrophy.

To explore the potential reasons for the decreasing trend in ESCC detection between 2003 and 2018, we used a dataset of 3605 subjects for whom all eight variables were available (ie, age, usual alcohol intake, alcoholic beverage most frequently consumed, cigarette smoking, BMI, chronic atrophic gastritis, *ALDH2* genotype, and *ADH1B* genotype). The ORs for the year of endoscopic screening were calculated using a multiple logistic analysis with the eight adjusted variables added in a step‐by‐step manner (ie, adjusted for age; age and usual alcohol intake; age, usual alcohol intake, and alcoholic beverage most frequently consumed; and so on). Any substantial change in the OR after the addition of a new adjusted variable would mean a strong influence of the adjusted variable on the decreasing trend in ESCC detection. However, when ESCC detection during the 2003‐2007 period was used as a reference, the ORs for the 2008‐2012 period (ORs = 0.40‐0.47) and the 2013‐2015 period (0.31‐0.38) essentially did not change after the addition of any of the adjusted variables.

## 4 DISCUSSION

The detection rates of ESCC (3.5%), HNSCC (1.1%), and GA (1.1%) were high during the initial screening of the alcohol‐dependent patients. The rate of GA detection has gradually decreased over time, while the rate of ESCC has decreased over the past decade.

Generations born before 1950 have a prevalence of *H pylori* infection of more than 60%. A drastic decline in prevalence occurred thereafter, reaching around 30% for subjects born during the 1970s.^10^ This reduction was mainly due to sanitary improvements during early childhood. Among the subjects of the 1993‐2007 screening, 35.8% had been born after 1950, while among the subjects of the 2008–2018, 73.5%. The reduction in *H pylori* infection is probably the main reason for the decrease in GA detection over time.

Technical improvements in endoscopes and a growing understanding of the endoscopic findings of early ESCC and HNSCC have enabled early cancer detection.^21^, ^22^ These improvements may explain the slight increase in the detection rates of ESCC and HNSCC during the 1993‐2007 period. We have routinely applied NBI inspections of the esophagus and head and neck region in conjunction with esophageal iodine staining since 2009. A meta‐analysis has shown the adequate ability of NBI to diagnose early ESCC with a high sensitivity similar to that achieved using esophageal iodine staining.^23^ The reduction in ESCC detection during the past decade is all the more puzzling in consideration of such diagnostic improvements.

ESCC, HNSCC, and GA were frequently concurrent, suggesting a common background underlying these cancers. The inactive heterozygous ALDH2*1/*2 genotype posed strong risks for ESCC, HNSCC, and GA. This was consistent with the results of earlier reports.[1, 2, 3, 4, 5, 6, 24 Acetaldehyde can cause the formation of acetaldehyde‐DNA adducts^25^ and DNA double‐strand breaks leading to chromosome rearrangements,^26^ and inhibition of the enzymes involved in DNA repair.^27^ The International Agency for Research on Cancer has classified “acetaldehyde associated with alcohol consumption” as a Group 1 human carcinogen.^28^

A meta‐analysis of seven studies has shown a 1.94‐fold higher risk of ESCC among people with gastric atrophy.^29^ This study demonstrated high ORs for ESCC as well as GA among patients with severe CAG. We previously reported that the prevalence of *H pylori* infection was similar between Japanese alcohol‐dependent men with and without ESCC.^6^ The positive association between ESCC and CAG can be explained by mechanisms that are common to both ESCC and CAG development. This association might be a causal relationship. Oral microflora form acetaldehyde from ethanol and contribute to high acetaldehyde levels in the saliva after ethanol ingestion.^30^, ^31^ The saliva containing microflora and acetaldehyde is transported from the mouth to the esophagus and stomach. Oral bacterial overgrowth is common in the stomachs of alcohol‐dependent patients.^32^ The overgrowth of oral bacteria in an achlorhydric stomach environment can explain the human experimental finding that ALDH2 deficiency and proton pump inhibitor (PPI) treatment markedly increased the acetaldehyde levels in the gastric juice after intragastric ethanol administration.^33^ The numbers of total gastroesophageal reflux and non‐acid reflux episodes were relatively high in ESCC patients.^34^ Among drinkers with CAG, the reflux of acetaldehyde‐rich gastric juice may increase the risk of ESCC. Acid‐suppressive drugs, histamine 2‐receptor antagonists and PPIs are frequently administered for gastroesophageal reflux disease (GERD) or dyspepsia and for protection against non‐steroidal anti‐inflammatory drug‐induced peptic ulcers. A meta‐analysis of 11 observational studies has shown that acid‐suppressive drug use is associated with an increased risk of gastric cancer.^35^ If pharmacologically induced gastric hyposecretion is associated with increased risk for ESCC as well as GA in high‐risk drinkers, the increasing trend in acid‐suppressive drug use may have modified the cancer incidence trends; future evaluation of this issue is warranted.

Oral *Streptococcus* species are a major component of the esophageal microbiota.^36^ These species express multiple ADHs but do not express functional ALDH.^37^ A significant correlation between *Streptococcus anginosus* and carcinogenesis in the upper aerodigestive tract and stomach has been reported,^38^, ^39^ and the ratio of *Streptococcus anginosus* to oral bacteria was extremely high in the saliva of Japanese alcohol‐dependent men.^40^ Asian studies have demonstrated positive associations between excessive drinking and GERD or short segmental Barrett's esophagus.^41^, ^42^ Antiseptic acid reflux alters the esophageal microbiota in subjects without CAG. The alteration of esophageal microbiota, specifically an enrichment of Gram‐negative species, has been reported in patients with GERD or Barrett's esophagus.^36^ The reduction in ESCC detection during the past decade might be partly attributable to the reduction in *H pylori* infection and the subsequent alterations of microbiota and acetaldehyde exposure in the esophagus.

The most frequently consumed alcoholic beverages have changed over time. However, favorite beverage choices were not associated with the ORs for any type of cancer. The number of pack‐years decreased progressively by 7.5 pack‐years during the study period. A 10 pack‐year increment yielded a 6% increase in the OR for ESCC. A low BMI has been reported to be a risk factor of ESCC.^3^, ^43^ The BMI increased progressively by about 1 kg/m^2^ among the alcohol‐dependent men during 1993‐2018, yielding a 7% reduction in the OR for ESCC. The ORs for ESCC according to the screening periods, using the 2003‐2007 period as a reference, were calculated by adding adjusted variables including age, drinking and smoking habits, BMI, CAG, and *ALDH2* and *ADH1B* genotypes in a step‐by‐step manner. However, the ORs for the 2008‐2012 and 2013‐2015 screening periods essentially did not change after the addition of any of the adjusted variables, suggesting that unidentified risk factors for ESCC have been disappearing in this population during the recent decades. The age‐adjusted mortality rate for esophageal cancer was consistent at around 10 per 100 000 men between 1975 and 2005 in Japan, but this rate has been gradually decreasing since then, reaching 7.7 per 100 000 men in 2015.^44^ The reasons for the reduction in ESCC detection might not be specific to the alcohol‐dependent population in Japan. These trends in Japan might be also linked to global trends of decreasing incidence of male ESCC during recent decades.^45^ Changes in the prevalence of tobacco smoking and alcohol consumption may have contributed to global trends.^45^ However, the present declining trend in the incidence of ESCC in an alcohol‐dependent population warrants future research to identify other potential causes.

We did not observe any effects of recent alcohol consumption on cancer detection. This result might be related to the homogeneity of the study population with regard to alcohol dependence. It would be more relevant to study the effects of alcohol consumption for a longer period. Another limitation was that factors such as *H pylori* infection; oral, esophageal, and gastric microbiota; oral hygiene; and dietary habits including the intake of fruit and vegetables, salt, and high‐temperature beverages were not examined, and all of these factors have considerable impacts on carcinogenesis in the upper aerodigestive tract and stomach.^15^, ^46^, ^47^, ^48^

In conclusion, the endoscopic screening of alcohol‐dependent men yielded high detection rates of ESCC, HNSCC, and GA during 1993‐2018. The detection rates of ESCC as well as those of GA have markedly decreased during the past decade. Several risk factors for these cancers were identified, but the confounders cannot fully explain the reduction in ESCC detection.

## CONFLICT OF INTEREST

Nothing to report.
